# Proteome scanning to predict PDZ domain interactions using support vector machines

**DOI:** 10.1186/1471-2105-11-507

**Published:** 2010-10-12

**Authors:** Shirley Hui, Gary D Bader

**Affiliations:** 1 Donnelly Center for Cellular and Biomolecular Research, Banting and Best Department of Medical Research, University of Toronto, Toronto ON, Canada; 2 Department of Molecular Genetics, University of Toronto, Toronto ON, Canada

## Abstract

**Background:**

PDZ domains mediate protein-protein interactions involved in important biological processes through the recognition of short linear motifs in their target proteins. Two recent independent studies have used protein microarray or phage display technology to detect PDZ domain interactions with peptide ligands on a large scale. Several computational predictors of PDZ domain interactions have been developed, however they are trained using only protein microarray data and focus on limited subsets of PDZ domains. An accurate predictor of genomic PDZ domain interactions would allow the proteomes of organisms to be scanned for potential binders. Such an application would require an accurate and precise predictor to avoid generating too many false positive hits given the large amount of possible interactors in a given proteome. Once validated these predictions will help to increase the coverage of current PDZ domain interaction networks and further our understanding of the roles that PDZ domains play in a variety of biological processes.

**Results:**

We developed a PDZ domain interaction predictor using a support vector machine (SVM) trained with both protein microarray and phage display data. In order to use the phage display data for training, which only contains positive interactions, we developed a method to generate artificial negative interactions. Using cross-validation and a series of independent tests, we showed that our SVM successfully predicts interactions in different organisms. We then used the SVM to scan the proteomes of human, worm and fly to predict binders for several PDZ domains. Predictions were validated using known genomic interactions and published protein microarray experiments. Based on our results, new protein interactions potentially associated with Usher and Bardet-Biedl syndromes were predicted. A comparison of performance measures (F1 measure and FPR) for the SVM and published predictors demonstrated our SVM's improved accuracy and precision at proteome scanning.

**Conclusions:**

We built an SVM using mouse and human experimental training data to predict PDZ domain interactions. We showed that it correctly predicts known interactions from proteomes of different organisms and is more accurate and precise at proteome scanning compared with published state-of-the-art predictors.

## Background

Many protein-protein interactions in eukaryotic signalling systems are mediated by conserved modular protein recognition domains, which are organized in different ways to form larger proteins. The **P**SD95/**D**lgA/**Z**o-1 (PDZ) domain is a protein recognition domain that is found in increasing abundance in yeast to metazoans with 250 encoded in the human genome [1]. They are often found in scaffolding proteins and interact with ion channels, adhesion molecules, and neurotransmitter receptors in signalling proteins to maintain cell polarity, facilitate signal coupling and regulate synaptic development [2]. Furthermore, studies have shown that the disruption of PDZ domain mediated interactions are associated with diseases such as human papillomavirus, cystic fibrosis and schizophrenia [3-5].

The PDZ domain consists of 80-90 amino acid residues folded into five to six β strands and two α helices. Canonical interactions occur through the recognition of hydrophobic C terminal tails of target proteins binding in a groove formed between strand β2 and helix α2. Early studies grouped PDZ binding specificity into two classes focusing on residues at position 0 and -2 of the ligand [6] (position numbering counted backwards from the 0 C terminal position). However, it is now known that the PDZ binding pocket can interact with, and is highly specific to, as many as seven target C terminal residues enabling the recognition of a diverse set of ligands [7,8]. A wealth of knowledge about PDZ domain interactions is now available from various sources. Some focus on select family members while recent high throughput experiments study many domains from across the entire family [7-9].

The biological importance of PDZ domains, their simple modes of target recognition and the availability of experimentally determined interactions have prompted the development of PDZ domain interaction prediction methods by multiple groups. Such methods are based on established techniques, which have been used with success to predict interactions for SH2 and SH3 domains, protein serine-threonine kinases and major histocompatibility complex (MHC) molecules [10-14]. Position weight matrices (PWMs) contain in each cell the probability of observing an amino acid at a given ligand position and are commonly used to compute a score describing the binding preference of a PDZ domain for a given peptide. Tonikian et al., used PWMs to predict human PDZ interactions and identify viral proteins that mimicked domain specificities [7]. Stiffler et al., developed a variant of the PWM that contained weights describing the relative preference for amino acids at positions in the ligand compared to the other domains they modelled [9]. Another method by Eo et al. used a machine learning method called a support vector machine (SVM) to predict PDZ domain interactions, though limited to those involving G coupled proteins [15]. While these methods can predict PDZ domain interactions, their common limitation is that they were trained to ideally predict interactions for specific or limited subsets of PDZ domains. Recently, Chen et al., used an additive model to predict interactions for the entire PDZ domain family using data from Stiffler et al. [16]. They also demonstrated the predictor's ability to predict mouse genomic interactions and to a lesser extent genomic interactions in other organisms. This predictor was validated on a limited data set, thus it is not clear if it can be used to accurately and precisely predict interactions given a large set of possible interactors.

A practical application of a reliable PDZ domain interaction predictor would be to use it to scan the proteomes of organisms for potential binders of PDZ domains. The results would help direct future experiments to increase the coverage of current PDZ domain interaction networks and expand our knowledge of the roles that PDZ domains play in different biological processes. In this paper we present a primary sequence based predictor of genomic interactions involving PDZ domain family members using a SVM. Unlike published predictors, our SVM is trained using data from two independent high throughput studies using protein microarray and phage display technologies, which makes it more general. Since the phage display data consists of only positive interactions, we have overcome a major issue, which has up to now prevented its straightforward use for predictor training. We addressed this by developing a method for the generation of artificial negative interactions from data consisting of positive interactions only. This method generates more biologically meaningful negatives compared to other commonly used methods that use randomization or shuffling. Through independent testing with published genomic data sets, we showed the SVM's ability to accurately predict interactions in multiple organisms [16]. We then used the SVM to scan human, worm and fly proteomes to predict binders for different PDZ domains. We validated the predictions using known genomic interactions from PDZBase and protein microarray experiments [16,17]. Finally a comparison of proteome scanning performance, which depends on minimizing the number of false positives generated, showed the SVM's improved accuracy and precision compared to published predictors. Predicted interactions made by our SVM matched a significant number of known protein-protein interactions and were enriched in known and novel biological processes, suggesting that many more predictions are likely to be correct.

## Methods

### Training Data

We trained our predictor using data from mouse protein microarray and human phage display experiments [7,9]. Interactions were collected in the form of domain-peptide sequence pairs, where domains were represented by their binding site and peptides were five residues in length. For both mouse and human PDZ domains, we omitted those whose binding site did not align well with other PDZ domains [16]. Human domains that lacked adequate data (less than 10 interactions), or were difficult to generate artificial negative interactions for, were also not used. This left 82 out of 85 mouse and 31 out of 54 human PDZ domains. Since phage display data may contain non-genomic interactions and we were interested in building a genomic predictor, we filtered the human phage display data to create a data set enriched in genomic-like interactions. First, an interaction was considered to be genomic-like if the last four residues of the interacting peptide matched a human protein tail (defined by genome assembly Ensembl:GRCh37.56), otherwise it was defined as non genomic-like. Then, domains were categorized as genomic-like, non genomic-like, dual or non-specific, depending on the number of unique genomic-like or non genomic-like interacting peptides they bound to (Table 1). To enrich for genomic-like interactions we did not use any data from non genomic-like domains and removed all non genomic-like interactions from the dual domains. Domains with less than 10 unique genomic-like peptides after this filtering were not used. Finally, data from genomic-like and non specific domains (that had a combined total of ≥10 peptides) were used without any filtering. This resulted in a small number of non genomic-like interactions being included, but allowed us to increase the amount of phage display data usable for training. In total, data for 20 human and 82 mouse domains were used for training (Table 2). Please see additional file 1: Supplementary Information for more details about how the data sets were created.

**Table 1:** Domain category definitions based on the number of unique interacting genomic-like and non genomic-like peptides

Category	# Unique genomic-like peptides	# Unique non genomic-like peptides
Genomic-like	≥ 10	< 10

Non genomic-like	< 10	≥ 10

Dual	≥ 10	≥ 10

Non specific	< 10	< 10

**Table 2:** Summary of the training data

		Domains		Interactions	
**Organism**	**Source**	**# Pos**	**# Neg**	**# Pos**	**# Neg**

Mouse	Protein microarray	82	72	643	1324

Human	Phage display	20	-	363	-

Human	Artificial negatives	-	20	-	745

	Total	102	92	1006	2069

### Artificial negative interactions for phage display

Since we considered the prediction of PDZ domain interactions as a binary problem (i.e. binds or does not bind), training an effective predictor required both positive and negative interaction data. We generated artificial negative interactions for the human phage display data since they only contained positive interactions. Based on previous research the proper selection of artificial negatives is important for successful predictor training and evaluation [18,19]. Random and shuffled peptide sequences have been commonly used, but since these negatives do not resemble real sequences, they have been shown to produce predictors with lower accuracy when predicting real negative interactions [19]. We generated artificial negative interactions for training based on positive interactors (peptide ligands) modelled using PWMs. Therefore a PWM for a given PDZ domain was used to select likely negative interactors for that domain from a set of unique real interactors for all domains. For a given domain, negative interactors are those peptides with low PWM scores and low redundancy with other selected peptides. We set the score cut off to be the minimum score among all the PWM scores computed for the positive interactors (see additional file 1: Supplementary Information for more details). For the 20 human phage display domains, a total of 745 artificial negative interactions were generated (Table 2). The number of positive and negative training interactions was balanced using a weighted cost support vector machine.

### Primary sequence based feature encoding

Each domain-peptide sequence pair was encoded as a vector of numeric values representing features of a positive or negative interaction. Values were scaled to lie between 0 and 1 [20]. We used the 'contact map' encoding method described in Chen et al. A contact map contains information about contacting residues in the domain binding site and peptide derived from a protein structure of a PDZ domain complexed with a peptide ligand [16]. In total, 16 domain binding site positions found to be in contact (< 5.0 angstroms) with the last five peptide positions were used, based on the 3D structure of mouse α1-syntrophin in complex with a heptapeptide. This corresponded to 38 contacting domain and peptide position pairs. Each amino acid residue pair was numerically encoded as a binary vector of length 400 representing a 20 × 20 binary matrix to capture all possible amino acid pairs. The final encoding consisted of a binary vector of size 15200 (38 × 400). Contact maps for other domains were constructed via a multiple sequence alignment [16].

### Support vector machine

A support vector machine is a machine learning method that makes binary predictions [21,22]. Given interaction training data (**x_1_**,*y_1_*),...,(**x*_m_***,*y_m_*) where **x**_i _is a feature vector for domain d_i _and peptide p_i _and *y *is a class label such that *y_i _*= {-1, +1}, a binary predictor assigns a class label of +1 if a given interaction feature vector encodes a positive interaction or -1 otherwise. SVMs evaluate the following decision function to make binary predictions:

f(x)=sgn(w•x+b)

where sgn(0) = +1, otherwise -1. The margin *w *and bias term *b *describe a maximum margin hyperplane separating positive and negative training points and are solutions to the following optimization problem:

$$\begin{array}{l} W\left(\alpha \right)=\sum_{i=1}^{m}\alpha_{i}-\frac{1}{2}\sum_{i,j,=1}^{m}\alpha_{i}\alpha_{j}y_{i}y_{j}K\left(x_{i},x_{j}\right)\\ \text{subject to}\leq \alpha_{i}\leq C\text{ for all }i=1,...,m,\text{ and }\sum_{i=1}^{m}\alpha_{i}y_{i}=0 \end{array}$$

where K (**x_i_, x_j_**) can be regarded as describing the similarity between two feature vectors, α's are Lagrange Multipliers and C is a cost parameter that penalizes training errors. The radial basis function (RBF) kernel was used here and is defined as:

$$K\left(x_{i},x_{j}\right)=e^{-\gamma ||x_{i}-x_{j}|{|}^{2}}$$

Locally optimal values for γ and C were determined using grid search. We used weighted costs according to C^+ ^= (n^+^/n^-^) C^-^, where n^+ ^is the number of positive training interactions and n^- ^is the number of negative training interactions. LibSVM was used to build the SVM [23].

### Predictor performance evaluation

Multiple cross validation strategies were used to estimate the SVM's ability to extrapolate to unseen interaction data. We used 10 fold cross validation by partitioning the training data into 10 randomly selected interaction sets, independently holding out each set for testing against a predictor trained using the remainder of the data, and averaging the performance across all 10 runs. For comparison purposes, we also followed the procedure of Chen et al. [16], to estimate the predictor's ability to generalize to different unseen data by holding out 8% of the domains, 12% of the peptides and both 8% of the domains and 12% of the peptides and testing on the rest, again repeated 10 times.

We compared different predictors, through testing using independent genomic data sets in worm, fly, mouse and human collected from different sources (Table 3). In particular, we used data from Chen et al. [16], which included interactions from protein microarray experiments for fly, worm and mouse orphan domains. Mouse orphan domains were those from the original mouse protein microarray experiment that did not interact with any of the peptides tested. In Chen et al., a subset of interactions involving these domains were retested using fluorescence polarization to identify false negatives which were then corrected to be positive interactions [16]. We also used known human interaction data from PDZBase [17].

**Table 3:** Summary of data for independent genomic testing and prediction validation

		Domains		Interactions	
**Organism**	**Source**	**# Pos**	**# Neg**	**# Pos**	**# Neg**

Fly	Protein microarray	7	7	34	106

Worm	Protein microarray	6	6	59	88

Mouse (Orphan)	Protein microarray	11	19	52	74

Human	PDZBase	13	-	38	-

We computed the following statistics to measure predictor performance:

• True positive rate (TPR) or Recall: #TP/#P

• False positive rate (FPR): #FP/#N

• Precision: #TP/(#TP + #FP)

• F1 Measure: 2 (Precision × Recall)/(Precision + Recall)

where #TP is the number of true positives, #P is the number of positives, #FP is the number of false positives, #N is the number of negatives. The overall performance was summarized by computing the area under the ROC and PR curves (AUCs) [24,25].

## Results

### Estimating support vector machine performance

**Figure 1:**
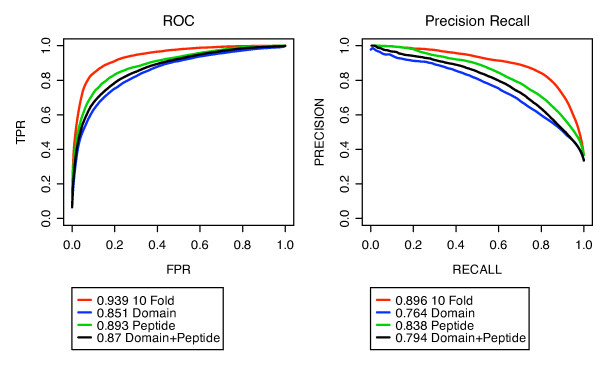
**SVM performance estimation using cross validation**. SVM performance measured using 10 fold (red), leave 12% of domains out (blue), leave 8% of peptides out (green), leave 12% of domains and 8% of peptides out (black) cross validation.

**Figure 2:**
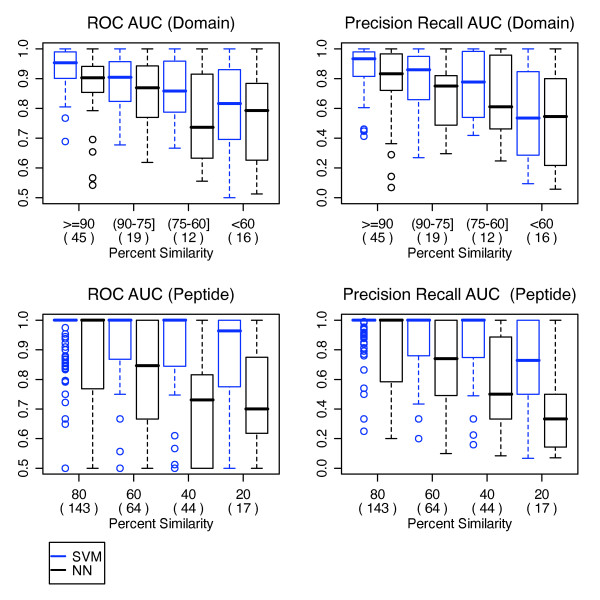
**SVM performance dependence on testing and nearest training neighbour sequence similarity**. Using leave one domain out cross validation (top), domain specific ROC and Precision/Recall AUC scores for SVM (blue) and nearest neighbour predictor (black) were grouped according to a given testing domain's similarity to its nearest training neighbour. The same was done for peptides using leave one peptide out cross validation (bottom). The similarity between two domains was calculated as the percentage of matched residues between their binding site sequences. The similarity between two peptides was calculated as the percentage of matched residues. Numbers in parentheses indicate the number of domains or peptides in each boxplot.

The SVM achieved high AUC scores from multiple cross validation testing. The highest ROC and PR AUCs of 0.939 and 0.896 respectively were obtained when 10% of interactions were held out for testing. For tests that involved holding out all interactions for a given domain, the AUC scores were lower. In particular, the leave 12% of domains out test yielded ROC and PR AUC scores of 0.851 and 0.764 and the leave 12% domain and 8% peptides out yielded ROC and PR AUC scores of 0.87 and 0.794 (Figure 1). In contrast, the leave 8% peptides out yielded higher ROC and PR AUCs of 0.893 and 0.838. This suggests that the SVM's ability to accurately predict a given test domain depends on its level of similarity to the training domains. To determine the degree of this dependency we performed leave one domain out cross validation and divided the AUC scores according to the binding site similarity of the held out domain to that of its nearest training neighbour. We repeated this using a simple nearest neighbour predictor (NN) and compared the results. The results showed that, indeed, the SVM achieves a higher performance for domains that are more similar to the training set. The SVM was on average better than the nearest neighbour method for testing domains with over 60% sequence similarity to their nearest training neighbour (Figure 2 top). Presumably, this means the SVM learned non-trivial patterns in the data features instead of simply indentifying similarities in the sequences as the NN predictor did. For tested peptides, this dependence was not as apparent, which indicates that the SVM's performance is more dependent on domain sequence similarity than peptide sequence similarity (Figure 2 bottom). For more details about nearest neighbour predictor see additional file 1: Supplementary Information.

### Performance evaluation on a series of independent tests across organisms

We next validated our choice of data and methods for three major parameters affecting predictor performance: training data, feature encoding and artificial negatives. We examined each parameter independently by comparing our SVM to other SVMs built using different values for the parameter of interest while holding the other two parameter values fixed. Predictor performance was assessed using data for mouse, worm and fly from independent protein microarray experiments, which all contain positive and negative interactions [16].

### Genomic-like phage display training data

**Figure 3:**
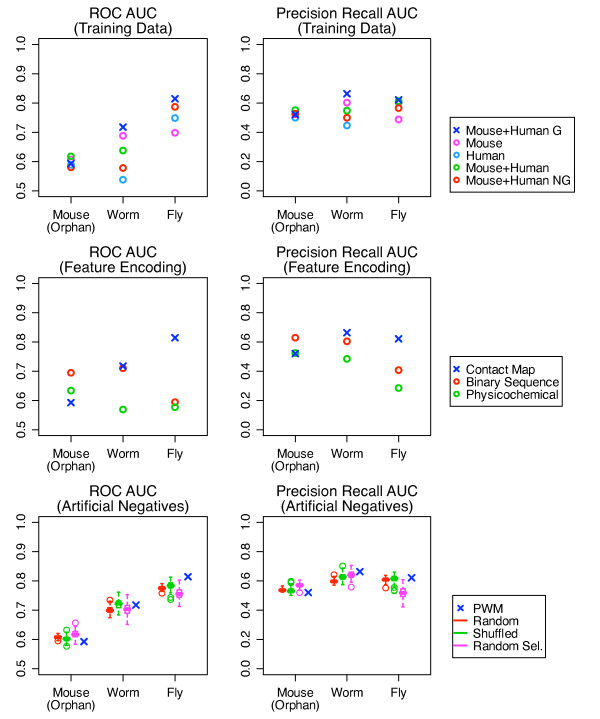
**Comparison of independent genomic test performance of different SVMs**. Blue × denotes data or method used by our SVM in all panels. (Top Row) A comparison of SVMs trained using data from one experiment: mouse from Chen et al. (magenta) or human from Tonikian et al. (light blue), from two experiments: mouse and human (green) and from two experiments with data enriched in genomic-like or non genomic-like human data: mouse and genomic-like human (blue) and mouse and non genomic-like human (red). (Middle Row) A comparison of SVMs trained using data encoded using different feature encodings: binary sequences (red), physicochemical properties (green), contact map (blue). (Bottom Row) A comparison of SVMs trained using different methods for generating artificial negatives for phage display: random peptides (red), shuffled peptides (green), randomly selected peptides (magenta), PWM selected peptides (blue). One hundred different SVMs trained using different random, shuffled and randomly selected peptides were built.

We first validated our use of mouse protein microarray and human genomic-like phage display data for training. We compared our SVM to those built using data from single experimental data types (mouse/protein microarray or human/phage display), both experimental data types (mouse/protein microarray and human/phage display) and both experimental data types but with human phage display data enriched in genomic-like or non genomic-like interactions. For all SVMs, contact map features were used to encode the data and PWMs were used to generate artificial negatives. A comparison of predictor performance showed that our SVM was better than the other predictors for the worm and fly tests (Figure 3). All predictors had lower scores for the mouse orphan test. To explain the latter observation, for each test we computed the binding site similarity of each testing domain to its nearest training neighbour. We found that the mouse orphan domains were on average 65% similar to their nearest training neighbours, while the worm and fly testing domains were on average 80% and 87% similar to their nearest training neighbours respectively. Therefore the observed pattern of performance was consistent with our earlier observation that predictor performance decreased as the similarity between testing domains to their nearest training neighbours decreased. These results validate our use of both mouse protein microarray and human genomic-like phage display interactions for SVM training.

### Contact map feature encoding

We next validated our choice of using the contact map feature encoding. We compared our SVM to those built using binary sequence or physicochemical property-based encodings. All predictors used mouse protein microarray and human genomic-like training data and PWMs to generate artificial negatives. For the binary sequence encoding, binary vectors were created using a vector of length 20 with each element representing an amino acid and initially set to zero. A single residue was represented by placing a one in the position representing that residue. A binary vector was created for each residue in a domain-peptide interaction pair, with the final vector of length 20 aa × (length of domain binding site sequence + length of the peptide sequence). For physicochemical features, a vector of five real numbers describing over 500 different physicochemical properties for each amino acid residue was created for a domain-peptide interaction sequence [26]. Thus, final vectors were of length 5 × (length of the domain binding site sequence + length of the peptide sequence). The predictor performance comparison showed that except for the mouse orphan test, our SVM had the highest scores (Figure 3). We again attributed the low performance on the mouse orphan test to the dissimilarity of the test domains to the training domains. Those predictors with better mouse orphan test performance did not generalize to the worm or fly tests, supporting the conclusion that the mouse test is not ideal. These results indicate that the contact map feature encoding for SVM training is better compared to binary and physicochemical property based encodings.

### PWM selected negative interactions for phage display data

Finally, we validated the use of PWMs for generating artificial negatives for the phage display training data. We compared our SVM to those built using random, shuffled, and randomly selected artificial negatives. All predictors used mouse protein microarray and human genomic-enriched phage display training data encoded using contact map features. Random negatives were created using random residues concatenated into peptides of length five. Shuffled negatives were created by shuffling residues in the positive peptides. Randomly selected negatives were created by randomly selecting peptides from the same set of peptides used to select negatives in the PWM method. We created 100 different artificial negative data sets from the phage display data and measured the mean predictor performance. Over all the tests, the average SVM ROC and PR AUC scores were 0.71 and 0.60, respectively, which were slightly higher than the over all average ROC and PR scores for the other predictors (Figure 3). Specifically, the average ROC and PR scores were 0.70 and 0.58 for random negatives, 0.70 and 0.59 for shuffled negatives and 0.69 and 0.58 for randomly selected negatives. Although the scores were similar for all predictors within each test, the average ROC and PR scores for mouse, worm and fly tests showed that all predictors performed poorly for the mouse orphan test but were better for the worm test. For the fly test however the predictor using PWM negatives was in general better. This suggests that the PWM negatives are a reasonable choice for artificial training negatives with its importance for improving predictor performance more evident in cases where the testing domain is highly similar to the training domains.

### SVM prediction of PDZ domain interactions by proteome scanning

We used the SVM to scan the human proteome (defined by genome assembly Ensembl:GRCh37.56) [27] to predict binders for 13 human PDZ domains with available validation data in PDZBase. In total, 41,193 unique transcript tails of length five, out of 77,748 transcripts corresponding to 23,675 genes from the human proteome, were scanned. We also scanned the worm and fly proteomes (defined respectively by genome assemblies Ensembl:WS200.56 and Ensembl:BDGP5.13.56) [27] for binders for six and seven PDZ domains respectively, with known genomic interactions from Chen et al. [16]. For worm, 19,864 unique transcript tails of length five, out of 27,533 transcripts corresponding to 20,158 genes, were scanned. For fly, 14,691 unique transcript tails of length five, out of 21,309 transcripts corresponding to 20,158 genes, were scanned. In all cases, very few known genomic interactions (on average 2.2 human, 4.2 worm and 9.8 fly) were available for validation of the domains tested making accurate assessment of predictor performance difficult. Nonetheless, the results reported here serve as a reasonable performance estimate. SVM predictions are available at http://baderlab.org/Data/PDZProteomeScanning.

For human, over 85% of PDZBase interactions for 10 of the 13 human domains were predicted by the SVM. Of the three remaining domains, MAGI2-2 and MAGI3-1 had no PDZBase interactions correctly predicted, but these domains had only one known interaction each. Two other domains (PDZK1-1 and SNTG1-1) also had only one known interaction each however the SVM correctly predicted the single interaction for these domains. Further experimental validation and more detailed literature searches should be carried out to obtain a more reliable assessment of SVM performance for these domains. For the last domain (MLLT-4), only one out of six known interactions was predicted, however compared to the other domains tested, this domain was the most dissimilar to its nearest training neighbour with a similarity of 0.68. It also had no homologs in the training data making it a challenging test case.

For worm and fly, 25% and 37% of protein microarray interactions respectively were predicted. Although this is much lower than the human proteome scanning result, the false positive rates are both quite low at approximately 4%. In particular, in worm and fly, none of the known interactions were predicted for DSH-1 despite it having a reasonable number of known interactions (11 and 3 respectively) and being very similar to its nearest training neighbour (over 0.8). In fly, the SVM did not make any predictions for PAR6-1 even though it too was very similar to its nearest training neighbour (1.0). Through further analysis, we found that in each case, the nearest training neighbours DSH-1 and PAR6B-1 in mouse had only three and two training interactions respectively. This suggests the possibility that predictor performance might also depend on the abundance of nearest neighbour training data. However, a single exception to this is that the SVM did not predict any known interactions for PATJ-2, which had a reasonable amount of validation data (7 interactions) and was very similar to its nearest training neighbour (over 0.81), which also had adequate data (16 interactions). Thus, in general, the SVM is more likely to correctly predict interactions for domains that are well represented in the training data in terms of sequence similarity and interaction abundance.

### Comparison of predicted and experimental binding specificities

**Figure 4:**
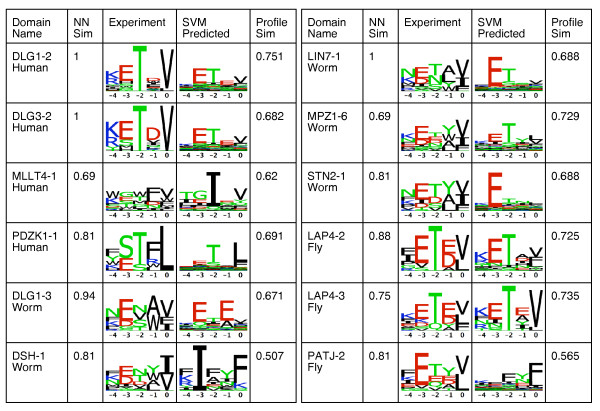
**Comparison of SVM predicted and experimental binding specificities**. A comparison of phage display determined and predicted PDZ domain binding specificities for the last five terminal binding positions visualized as sequence logos. For human, only domains with 10 or more peptides from phage display experiments by Tonikian et al. were compared. For worm and fly, domains with an adequate (five or more) number of peptides from protein microarray experiments by Chen et al. were compared.

Since known interactions are limited, we compared the predicted and experimental binding specificities to determine if the set of SVM predictions was consistent with their corresponding set of experimental binders, at a high level. Four of the human domains had adequate genomic-like binders from phage display experiments (10 or more), which were used to create PWMs to summarize their binding specificities. These were then graphically represented as sequence logos. For worm and fly, PWMs were created for five and three domains, respectively, that had five or more binders determined from protein microarray experiments. We then created PWMs using the corresponding predicted binders and computed the similarity between the predicted and experimentally determined binding specificities. The average PWM similarity was 67% and the predicted binding specificities corresponded to known PDZ domain binding classes I and II (Figure 4). Two domains (DSH-1 from worm, PATJ-2 from fly) had binding specificity similarities much lower than the average (less than 60%), however these results were not unexpected, given the poor results for these two domains shown above.

Although the experimental and predicted binding specificities were generally consistent, there were some discrepancies. For example the human phage display sequence logos show a clear preference for T at p-2 and V at p0 while this preference is not as strong for the predicted sequence logos. This is because phage display experiments only find optimal binders. However, such binders may not exist in the proteome, leading to the domain preferring a less optimal binder. This may be biologically advantageous as weak binders may allow for easier interaction regulation. To determine whether this was the case in our data, we scanned the human proteome with the optimal phage display PWMs and created genomic sequence logos with the top 1% of binders. The predicted sequence logos were all more similar to the genomic phage display sequences logos than they were to the optimal phage display sequence logos (see additional file 1: Supplementary Information Figure S2). Therefore, some discrepancies between experiment and predicted logos are not unexpected. Overall, these results show that the SVM predicted binding specificities are generally consistent with those that are experimentally determined.

### Protein-protein interaction support for predicted interactions

To provide additional support for our predictions, we calculated how many corresponded to known protein-protein interactions (PPIs). Specifically, we scanned the human proteome for potential binders for 213 human PDZ domains with known PPIs in the iRefIndex [28], which is a database integrating PPIs from different databases including BIND, BioGRID, CORUM, DIP, HPRD, IntAct, MINT. If the protein containing the given domain was found to interact with another protein whose C terminal tail matched the predicted binder, the prediction was considered to correspond to a known PPI. The SVM successfully predicted interactions corresponding to known PPIs for 75 of the 213 PDZ domains with an average of 19% of known PPIs successfully predicted per domain (see additional file 1: Supplementary Information Table S9). The number of PPIs successfully predicted per domain was significant (*p *< 0.05) for all but 19 domains. Significance testing was performed using Fisher's exact test, which asked whether the observed number of PPIs predicted for a given domain could be achieved at random. Since PDZ domain containing proteins may contain multiple PDZ domains, it is not possible to uniquely assign a PPI to a PDZ domain. This could result in erroneous false negative or true positive statistics for the above tests, thus they should be regarded as a rough estimate of predictor performance. There were not enough PPI data in iRefIndex to carry out the same analysis for worm and fly domains. SVM predictions are available at http://baderlab.org/Data/PDZProteomeScanning.

### SVM performance compared to published predictors

**Figure 5:**
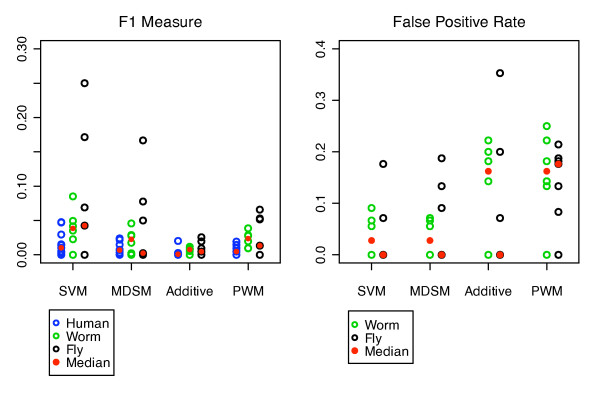
**Comparison of proteome scanning performances for SVM and published predictors**. A comparison of predictor performance evaluated using F1 measures and FPRs for 13 human (blue), 6 worm (green) and 7 fly (black) PDZ domains. Three different predictors were compared: MDSM, additive model and a PWM predictor. PDZBase interactions were used to validate human predictions. Protein microarray interactions from Chen et al. were used to validate fly and worm predictions. The median is denoted by the red circle. No FPRs were calculated for human predictions since there are no negative human validation interaction data. MDSM and the additive model were trained in their original publications using mouse protein microarray data only. The PWM predictor was trained using the same mouse and human data as the SVM.

Cross validation and a series of independent tests show that our SVM can accurately predict PDZ domain-peptide interactions, however, a major issue with most predictors used to scan a proteome is the generation of too many false positives. We thus compared the proteome scanning performance of our SVM and published prediction methods - the multidomain selectivity model (MDSM) by Stiffler et al. and the additive model by Chen et al. which are both state-of-the art and trained using mouse protein microarray data in their original publications [9,16]. We also developed an ensemble of PWMs, one per domain, built using the same data used to train the SVM. The PWM corresponding to the nearest training neighbour for a given test domain, as measured by binding site similarity, was then used to scan the proteome for the top 1% of PWM scoring binders. This predictor represented our baseline for comparison. We used the F1 measure to compare predictor performance since it summarizes the precision/recall performance of a predictor and is used in document retrieval where the recovery of relevant documents from a large number of possibilities is critical. For all predictors, the majority of F1 measures are low (less than 0.1). This is likely due to the high level of incompleteness in the benchmark used to validate the predictions. However, the results show that the SVM achieves a higher average F1 measure (0.037) than the other predictors demonstrating its improved accuracy and precision. In comparison, the average F1 measures were 0.02, 0.005 and 0.016 for the MDSM, additive model and PWM predictor respectively. For fly and worm domains, we computed the false positive rate and found it to be approximately 4% and substantially (over 4 times) lower than the FPRs of the other predictors (Figure 5).

The performance of the MDSM and SVM was close and the SVM's improved performance may be due to its use of a larger training data set (both phage display and microarray). To more directly compare these two predictors, we trained an SVM with only mouse microarray data and compared the performance. The results show that no predictor method is clearly better than the other. The MDSM's performance is not consistent as shown by the fly test results, which has similar testing and training data sets, and is expected to be an easy test (see additional file 1: Supplementary Information Figure S3 left). On the other hand, the performance of the SVM trained only using microarray data is more consistent, but has a higher false positive rate compared to the MDSM (see additional file 1: Supplementary Information Figure S3 right). These results suggest that our predictor performance improvement is likely due to our use of more training data. It may be possible to modify the MDSM method to accept phage display data as training, though the SVM method naturally accepts this data without method modification - a clear advantage in terms of flexibility. Overall, these results demonstrate the SVM's improved performance over other published predictors for proteome scanning of PDZ domain interactions. For more details about the predictors used for comparison please see additional file 1: Supplementary Information.

### Furthering our understanding of PDZ domains and the biological processes they mediate

To demonstrate how our predictions can be used to further our understanding of PDZ domains and the biological processes they mediate, we performed GO biological process term enrichment analysis of the predicted binders using the BiNGO (Biological Network Gene Ontology tool) software library [29]. The hypergeometric test was used to compute a *p*-value assessing the GO term enrichment for a given set of predicted genes. Multiple testing correction was performed using the Benjamini and Hochberg False Discovery Rate (FDR) correction. While we did not have enough information to perform the analysis with worm and fly, almost all human PDZ domain binder lists were statistically enriched (*p *< 0.05) for known PDZ domain processes such as ion transport and localization (see additional file 1: Supplementary Information Table S10). Interestingly, the biological process 'photoreceptor cell maintenance' was found enriched only among the predicted genes for the PDZ domain containing protein PDZK1-1. These genes include those that encode proteins associated with Usher (USH1G, USH2A) and Bardet-Biedl syndromes (BBS10); both are genetic human diseases of the cilia with wide ranging symptoms including retinal degeneration [30]. Although disruption of PDZ mediated interactions are known for Usher syndrome, such a disruption involving PDZK1-1 has not been reported for either. Since the validity of our predicted binders is supported by the successful prediction of known interactions in PDZBase and iRefIndex (1 out of 1 and 4 out of 24 respectively), with experimental validation, these potential PDZ domain mediated interactions may provide further insight into the molecular mechanisms underlying Usher and Bardet-Biedl syndromes.

## Discussion

We have presented a predictor, which can be used to more accurately and precisely scan proteomes of organisms for potential binders of PDZ domains. We focused on the application of proteome scanning. The results of our predictor can help prioritize biological experiments. In addition, since our predictions are predicted *in vitro *interactions, they can also be used as input to computational methods aiming to predict likely *in vivo *interactions by including multiple lines of evidence, such as co-expression and binding site conservation [31,32]. In both cases our predictions will be useful for substantially reducing the number of candidates that need to be considered for more focused analyses. Given the success of our proteome scanning results we also expect the predictor to perform well in organisms which are closely related to human, worm and fly.

An interesting result from our work is that binding site sequence information at contacting positions in the domain was the most effective feature encoding method among the ones we tried. The poor performance obtained by the other encoding methods (flatly representing binary sequence or physicochemical properties) suggest that by explicitly encoding contacting domain and peptide position pairs, sequence information need only be used to obtain good predictor performance. While we showed that this results in a predictor that relies to some degree on binding site sequence similarity, we also showed that this dependence only exists for the domain and not the peptide. We established a sequence similarity threshold of 60% for testing domains, which may act as a rough indicator of the limits of our predictor and can be used identify poorly characterized PDZ domains in current data sets.

The use of PWMs to generate artificial negatives was motivated by previous work that showed the importance of training with artificial negatives, which resemble real negative interactions. In one study, predictors were trained using random and shuffled negatives to show that this resulted in predictors with lower accuracy when real sequences were used for testing [18,19]. In other work, artificial negatives were generated by pairing proteins with different co localizations or randomly pairing proteins known to not interact. It was shown that this created a constraint on the distribution of the negatives making it easier for the predictor to distinguish between positive and negative interactions. This led to biased estimates of predictor performance when cross validation was used [18]. Since our PWM negatives were selected from peptides involved in real positive interactions, they are biological sequences and we expect their distribution to be closer to biologically meaningful interactions. We also believe that this results in a more realistic learning problem for the predictor and may reduce the bias in predictor accuracy estimation and benefit predictor performance in practice. However, we realize that PWMs may have high false positive rates due to limitations such as their inability to model dependencies between ligand positions. These shortcomings may be responsible for the modest improvement in independent testing performance between predictors trained using PWM generated and other negatives.

Although many of our proteome scanning predictions were validated using known interactions, the lack of a complete benchmark of genomic PDZ domain interactions contributes to our low F1 measures (most are less than 0.1). This may be addressed to some degree by using more validation data from experiments or literature searches, which we expect to help improve the accuracy of the F1 and FPR measurements. In the case of two fly domains LAP4-2 and LAP4-3, the SVM did achieve higher F1 measures of 0.17 and 0.25 respectively. The SVM predicted many known interactions but also predicted a very small number of fly proteins as potential binders (34 and 8 respectively). In general, the SVM made far less positive predictions than the other predictors, which raises the question of whether the SVM is simply more conservative (by making fewer predictions) or actually more precise (by making fewer and more accurate predictions) compared to other predictors. Again, this cannot be fully answered without more validation data, however the SVM's higher F1 and lower FPR scores are strong evidence supporting the latter case.

In genomic tests, predictor performance was consistently poor for the mouse orphan test, which consisted of domains that were highly dissimilar to the training domains. Based on our finding that predictor performance depends on the similarity between testing and training domains, this result was not unexpected. However, even if the similarity between testing and training domains is similar, predictor performance can still be poor. This was discovered while scanning the fly proteome for binders of PATJ-2. We found that the nearest training neighbour for this domain according to binding site sequence similarity did not correspond to its known human homolog, which was present in the training data. This highlighted a limitation generally faced by sequence based predictors: if the training domains best representing a given testing domain do not share similar sequence features, the correct binding specificity may not be properly learned. This may occur for two domains with structurally or physicochemically similar binding sites encoded with very different amino acid sequences. This may be the reason for the SVM's inability to predict any known interactions for PATJ-2. Exploring structural domain features useful for SVM training may determine if this is the case.

While our SVM performs better than published methods on proteome scanning, it can clearly be improved. One way to do this is to consider additional relevant features, such as information related to protein structure. For example, it has been shown that entropic and thermodynamic features of PDZ domain binding can vary considerably across PDZ domains and even for the same PDZ domain bound to different ligands [33,34]. Therefore, including dynamic features such as electrostatic or non-polar contributions between contacting residues may be used to help improve SVM performance. Another approach would be to use an SVM with a structure based kernel for PDZ domains. Indeed, recent work showed that an SVM using a structure based kernel was successful in the more general problem of predicting protein-protein interactions [35]. The main challenge for both these approaches is that 3D structures are not available for the majority of PDZ domains and homology modelling would be needed to increase the number of domains available for training and testing. A structure-based approach may also be used to generate more accurate biologically meaningful artificial negatives for training. Thus, until larger training datasets are available, we may require a combination of strategies to predict PDZ domain interactions, involving both sequence and structure-based methods, to maximize coverage and prediction performance. Nonetheless, here we have shown that sequence similarity is an important feature for accurately predicting PDZ domain interactions and it will be interesting to see how general this feature is for other domains.

## Conclusions

We describe a SVM for the prediction of genomic PDZ domain interactions. Our method uses training data from two independent high throughput experiments from mouse and human, for the first time, which improves performance. We showed that compared to published state-of-the-art predictors, our predictor can be used to more accurately and precisely scan proteomes for potential binders of PDZ domains. These predictions can be used to increase the accuracy and coverage of PDZ domain interaction networks and further our understanding of the roles that PDZ domains play in a variety of biological processes. Ideally, we would construct predictors like this one for all peptide recognition domains and use them to help map protein interactions in the cell.

## Availability and Requirements

Project name: PDZ Proteome Scanning

Project home page: http://baderlab.org/Data/PDZProteomeScanning

Operating systems: Platform independent

Programming language: Java 1.5

License: Source code is freely available under the GNU Lesser Public General License (LPGL).

## Authors' contributions

SH collected the data, developed and implemented the algorithm and drafted and revised this manuscript. GDB substantially contributed to all aspects of this project including conception and design and critically revised the manuscript. All authors read and approved the final manuscript.

## Supplementary Material

Additional file 1 **Supplementary Information**. HuiBader2010-BMC-SupplementaryInfo.pdf. This file contains more details about the data and methods discussed in this paper. Click here for file
