# Supplementary material for: Proteome scanning to predict PDZ domain interactions using support vector machines
Source: BMC Bioinformatics. 2010 Oct 12;11:507. doi: 10.1186/1471-2105-11-507 (PMC2967561; doi:10.1186/1471-2105-11-507)
Supplement: Additional file 1 — Supplementary Information. HuiBader2010-BMC-SupplementaryInfo.pdf. This file contains more details about the data and methods discussed in this paper. [file 1471-2105-11-507-S1.PDF]

Supplementary Information

Proteome scanning to predict PDZ domain interactions using support vector machines

Shirley Hui[1,2], Gary D. Bader [1,2,§]

[1] Donnelly Center for Cellular and Biomolecular Research, Banting and Best Department of Medical Research, University of Toronto, Toronto ON, Canada

[2] Department of Molecular Genetics, University of Toronto, Toronto ON, Canada

[§]Corresponding author

Email addresses:

SH: shirley.hui@utoronto.ca

GDB: gary.bader@utoronto.ca

Availability:

Project name: PDZ Proteome Scanning

Project home page: http://baderlab.org/Data/PDZProteomeScanning

Operating systems: Platform independent

Programming language: Java 1.5

License: Source code is freely available under the GNU Lesser Public General License (LPGL).

A. Optimization of SVM parameters

The RBF kernel parameter gamma and the SVM cost parameter C were optimized by performing a coarse two dimensional grid search over combinations of C ={2,4,6,8,10} and γ = {2,4,6,8,10}, with a finer grid search over combinations of C = {2,3,4,5,6} and γ = {3,4,5}. A 10 fold cross validation was used to evaluate the average ROC AUC score for each combination of γ and C. The parameters values yielding the predictor with the highest ROC AUC score were used. LibSVM was used to build the SVMs [1].

B. Constructing phage display data enriched in genomic-like or non genomic-like interactions

Categorization of human phage display domains

From the Tonikian et al. data set, 31 out of 54 human phage display domains were used to create data sets enriched in genomic-like or non genomic-like interactions [2]. A peptide was genomic-like if its last four residues matched a protein tail from the human proteome (Ensembl:GRCh37.56), otherwise it was non genomic-like. Depending on how many unique interacting genomic-like or non genomic-like peptides, domains were then categorized as genomic-like, non genomic-like, dual or non specific according to the definitions in Table S1. The categorized domains are listed in Table S2.

Table S1. The following table summarizes the domain category definitions used to identify genomic-like, non genomic-like, dual and non specific domains in the phage display data set. The number of unique genomic-like peptides is the number of unique peptides that match a human protein tail (based on the last four residues in the peptide).

Category	# Unique genomic-like	# Unique non genomic-like

	interactions	interactions
Genomic-like	≥ 10	< 10
Non genomic-like	< 10	≥ 10
Dual	≥ 10	≥ 10
Non specific	< 10	< 10

Table S2. The following table lists the categorization of human phage display domains based on the definitions in Table S1. The number of unique genomic-like peptides is the number of unique interacting peptides that match a human protein tail (based on the last four residues in the peptide).

Tonikian Domain Name	# Unique genomic-like peptides	# Unique non genomic-like peptides	Category
DLG3-2	11	7	Genomic-like
PTPN13-2	11	9	Genomic-like
DLG1-2	18	22	Dual
MPDZ-1	11	48	Dual
MPDZ-3	11	24	Dual
SHANK3-1	21	13	Dual
APBA3-1	4	13	Non Genomic-like
DVL2-1	4	10	Non Genomic-like
HTRA2-1	2	28	Non Genomic-like
MAGI3-3	3	12	Non Genomic-like
MPDZ-13	6	12	Non Genomic-like
MPDZ-2	2	24	Non Genomic-like
MPDZ-7	3	15	Non Genomic-like
PDLIM2-1	1	23	Non Genomic-like
PSCDBP-1	4	46	Non Genomic-like
PTPN13-4	2	12	Non Genomic-like
TJP1-1	5	19	Non Genomic-like
DLG1-1	4	5	Non Specific
DLG1-3	8	3	Non Specific
DLG2-3	6	3	Non Specific
DLG4-3	7	5	Non Specific
ERBB2IP-1	3	9	Non Specific
INADL-2	2	4	Non Specific
LRRC7-1	3	5	Non Specific
MAGI1-4	3	9	Non Specific
MPDZ-10	6	7	Non Specific
MPDZ-12	1	7	Non Specific

PDLIM4-1	2	8	Non Specific
SCRIB-1	8	2	Non Specific
SCRIB-2	2	3	Non Specific
SNTA1-1	6	4	Non Specific

Constructing human phage display enriched in genomic-like or non genomic-like interactions

For a data set enriched in genomic-like interactions, only non genomic-like and dual domains were pre-processed and used for training. From these domains, all non genomic-like interactions were removed. If doing so resulted in a domain with less than 10 unique genomic-like peptides, this domain was not used for training. Data from genomic-like and non specific domains (if they had ≥ 10 interactions in total) were used without any pre-processing. In total, 20 human domains were used for training. For a data set enriched in non genomic-like interactions, only genomic-like and dual domains were pre-processed. From these domains, all genomic-like interactions were removed. If doing so resulted in a domain with less than 10 unique interacting non genomic-like peptides, that domain was not used for training. Data from non genomic-like and non specific domains were used without any pre-processing. In total, 29 human domains were used for training in this case. Table S3 contains a summary of the genomic-like and non genomic-like phage display training data sets.

Table S3. The following table summarizes the human phage display data used for training. * denotes interactions used to create phage display training data enriched in genomic-like interactions. ** denotes interactions used to create phage display training data enriched in non genomic-like interactions.

Tonikian Domain Name	Total # genomic-like	Total # non genomic-like	Total # interactions

	interactions	interactions	
DLG3-2	16	12	28 *
PTPN13-2	14	10	24 *
DLG1-2	22 *	26 **	48
MPDZ-1	14 *	51 **	65
MPDZ-3	12 *	25 **	37
SHANK3-1	35 *	17 **	52
APBA3-1	4	14	18 **
DVL2-1	4	18	22 **
HTRA2-1	0	30	32 **
MAGI3-3	2	12	15 **
MPDZ-13	9	29	42 **
MPDZ-2	3	32	35 **
MPDZ-7	13	25	28 **
PDLIM2-1	3	40	41 **
PSCDBP-1	3	69	75 **
PTPN13-4	1	20	22 **
TJP1-1	6	24	39 **
DLG1-1	6	12	12 *,**
DLG1-3	10	13	13 *,**
DLG2-3	10	14	14 *,**
DLG4-3	9	16	16 *,**
ERBB2IP-1	7	33	33 *,**
INADL-2	3	11	11 *,**
LRRC7-1	3	26	26 *,**
MAGI1-4	3	12	12 *,**
MPDZ-10	8	16	16 *,**
MPDZ-12	2	11	11 *,**
PDLIM4-1	2	10	10 *,**
SCRIB-1	23	27	27 *,**
SCRIB-2	2	16	16 *,**
2SNTA1-1	7	11	11 *,**

Human phage display domains excluded from training

In total 23 domains were not used for testing. Five domains had less than 10 peptides in total and the binding site sequence alignments for 17 domains did not align well to other PDZ domains (i.e. had at least one gap). The domain MLLT4-1 was also not used since we could not predict any negatives for it. Table S4 contains a summary of the phage display domains, which were not used for training.

Table S4. The following table lists the PDZ domains that were not used for training and the reasons for exclusion.

Tonikian Domain Name	# Interactions	Reason for exclusion
INADL-3	8	< 10
INADL-6	7	< 10
LIN7A-1	6	< 10
PARD3-3	5	< 10
PTPN4-1	6	< 10
CASK-1	20	Gapped
HTRA1-1	14	Gapped
HTRA3-1	66	Gapped
MAGI1-2	48	Gapped
MAGI3-2	15	Gapped
MPDZ-4	4	Gapped
MPDZ-5	13	Gapped
MPDZ-9	26	Gapped
MPP6-1	17	Gapped
PDZK1-1	30	Gapped
PDZK1-2	8	Gapped
SCRIB-3	32	Gapped
SLC9A3R2-2	37	Gapped
TIAM1-1	8	Gapped
TIAM2-1	7	Gapped
TJP1-3	33	Gapped
TJP2-3	32	Gapped
MLLT4-1	116	No negatives predicted

C. Artificial negatives for phage display training data

Using the 20 human phage display profiles from the previous section, different negative interactions were generated using the following methods. The same number of negatives was generated for each method.

a. Random: Given a domain, negative peptides were created by generating sequences of random residues of length five.

b. Shuffled: Given a domain, negative peptides were created by shuffling the residues of positive binders.

c. Random Selection: All unique peptides from the positive training interactions were put into a list to create a pool of peptides. Given a domain, peptides were randomly selected from this list.

d. PWM: All unique peptides from the positive training interactions were put into a list to create a pool of peptides. Given a domain with a corresponding set of positive peptide sequences (representing positive interactions determined from phage display), the following steps were taken to determine low scoring and low redundancy artificial negatives:

    1. A PWM was built using the positive peptide sequences and the minimum PWM score amongst the positive peptides was set to be the cutoff.

    2. All unique peptides in the pool were scored with the PWM from step 1 and sorted in descending order according to PWM score. Walking down the sorted list, peptides were selected based on two criteria:

        i. Low scoring: the PWM score must be lower than the cutoff

        ii. Low redundancy: The similarity of the peptide to peptides already selected must be below the redundancy threshold (in our case it must have less than three residues in common with negative peptides already selected). The choice of our redundancy threshold is explained in detail below.

D. Choosing the number of peptides in common to remove for peptide redundancy reduction

When selecting negative peptides in step 2 of the previous section, only peptides with less than three residues in common with those already selected were used. We optimized this redundancy threshold by building different SVMs trained using artificial negatives selected using different redundancy thresholds (1,2,3,4,5). For example, using a low threshold (less than one residue in common) would allow fewer but a more diverse set of negatives to be selected than using a higher threshold (less than five in common) which would allow a greater number but an overall less diverse set of negatives to be selected. The SVM with the highest ROC and PR AUCs was used and corresponded to a redundancy threshold of three (Figure. S1).

Figure S1. (Top row) ROC AUC comparison for predictors trained using data with different levels of peptide redundancy. (Bottom row) PR AUC comparison for predictors trained using data with different levels of peptide redundancy. Black coloured bars indicate the number used for our final SVM.

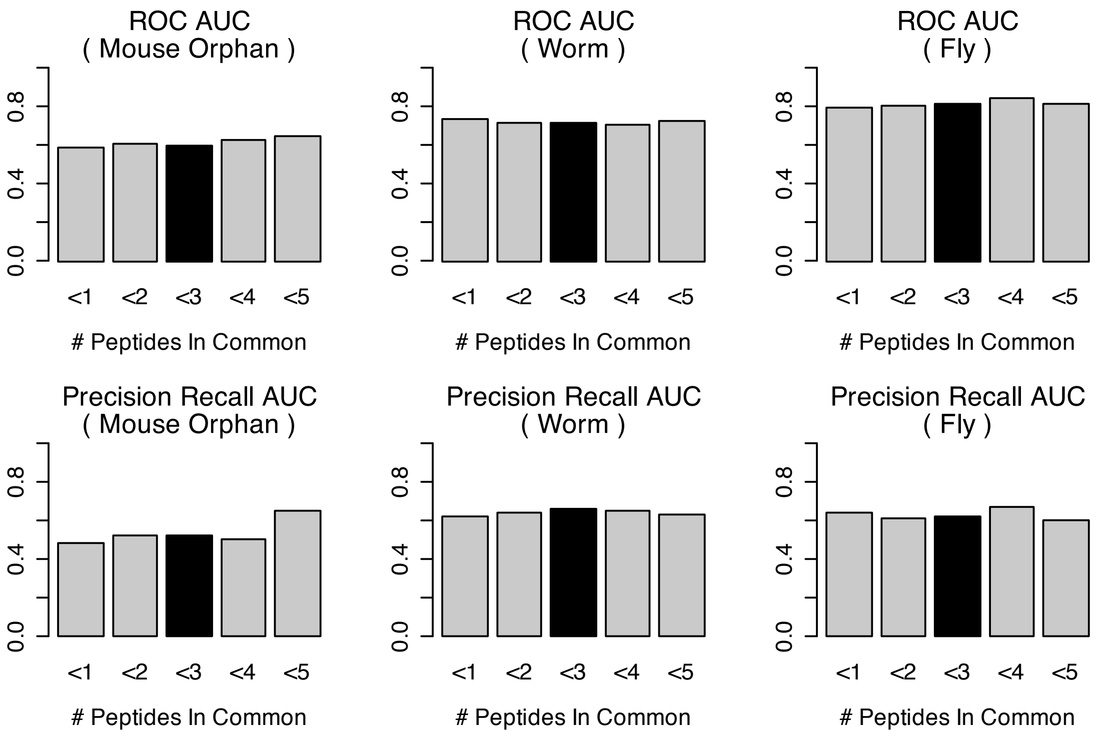

E. Implementation details for published and commonly used predictors

Several predictors for the prediction of PDZ domain interactions were used in this paper are discussed in more detail here. Binding site refers to the 16 domain sequence positions found to be in contact with the peptide ligand as described by Chen et al. [3].

Position Weight Matrix

Position weight matrices (PWMs) for each training domain were built using their known binders and represented their binding preferences. Thus the cells of the position weight matrices contain the log probability of each residue at each of the positions in the binding peptide. Since some amino acids occur more frequently than others, this bias is corrected for by dividing the PWM residue frequencies by their expected frequencies using the NNK codon set (where N represents a 25% mix each of adenine, thymine, guanine, and

cytosine nucleotides; and K represents a 50% mix each of thymine and guanine nucleotides) [4]. To avoid negative infinity values in the PWM, any residues with a frequency of zero are assigned the pseudocount of 0.01. The binding preference of a domain for a given peptide sequence is then computed by summing the weights in the matrix corresponding to each residue and position in the given sequence. If the score is above a specified cut off, the peptide is predicted to bind otherwise it is predicted to not bind. Using the nearest neighbour PWM of a given test domain (as determined by binding site sequence similarity), a list of peptides is evaluated and ordered in descending order by PWM score. The top 1% of this ordered list is then predicted to be binders. In total, interactions for 82 mouse from protein microarray and 20 human domains from phage display experiments as described in the paper were used to build the PWMs.

Nearest Neighbour

A nearest neighbour (NN) predictor was built and determined whether or not a given interaction was positive or negative using a nearest neighbour criterion. The nearest neighbour criterion is evaluated by computing the Hamming distance between a test interaction and all other training interactions (where interactions are represented as a domain binding site–peptide sequence pair). The training interaction with the lowest distance is then set to be the test interaction's nearest neighbour. Thus if the nearest neighbour is a positive interaction, the test interaction is predicted to be positive, otherwise it is predicted to be negative. In total, interactions for 82 mouse domains from protein microarray and 20 human domains from phage display as described in the paper were used to build the NN predictor.

Multidomain Selectivity Model

This multidomain selectivity model (MDSM) was built by Stiffler et al. [5] and computes the individual binding preferences of a given peptide to each mouse PDZ domain represented in the model. For our purposes, the binding preference of a given peptide was computed using the model parameters corresponding to its nearest model domain as determined by the Hamming distance between the binding site sequences. A given peptide is predicted to be positive if the binding preference score is greater than a predetermined threshold (we used the parameter m = 5 according to the original publication). In total 74 mouse PDZ domains were modelled.

Additive Model

We used the model parameters as specified in the tutorial provided in the supplemental material of the original publication [3]. The value of tau used was -0.3978. In total, 82 mouse domains from the Stiffler et al. protein microarray experiment were used for training in the original publication.

F. Detailed summary of proteome scanning results

The following is a summary of the results of proteome scanning in different organisms using the SVM, MDSM, additive model and PWM predictor. Method is the name of the predictor used, Domain is the name of the domain that the proteome is being scanned for, NN Sim is the similarity of the scanning domain to its nearest training neighbour, Num predicted is the number of positive predictions made by the predictor, #TP is the number of positive predictions validated to be positive, #FP is the number of positive predictions that were validated to be negative, #Valid Positives is the number of positive validation interactions, #Valid Negatives is the number of negative validation interactions. Only

validation interactions involving genomic peptides (as defined by the Ensembl genome assemblies) were used.

Human

The human proteome was scanned to predict interactions for 13 human PDZ domains with available interactions from PDZBase [6]. In total, 41,193 unique transcript tails of length five out of 77,748 transcripts corresponding to 23,675 genes from the human proteome were scanned (defined by Ensembl:GRCh37.56 genome assembly) [7].

Table S5. The following table summarizes the human proteome scanning results for the SVM, MDSM, Additive and PWM predictors

Method	Domain	NN Sim	Num Predicted	#TP	#FP	#Valid Positives	#Valid Negatives
SVM	DLG1-1	1.0	283	2	0	2	0
SVM	DLG1-2	1.0	389	3	0	3	0
SVM	MPDZ-10	1.0	199	3	0	4	0
SVM	ERBB2IP-1	1.0	83	2	0	2	0
SVM	DLG3-2	1.0	389	1	0	2	0
SVM	LIN7B-1	1.0	422	1	0	2	0
SVM	DLG4-1	0.9375	223	2	0	2	0
SVM	DLG4-2	0.9375	294	2	0	2	0
SVM	PDZK1-1	0.8125	551	1	0	1	0
SVM	MLLT4-1	0.6875	36	1	0	6	0
SVM	MAGI3-1	1.0	1185	0	0	1	0
SVM	MAGI2-2	1.0	694	0	0	1	0
SVM	SNTG1-1	1.0	680	1	0	1	0
Method	Domain	NN Sim	Num Predicted	#TP	#FP	#Valid Positives	#Valid Negatives
MDSM	DLG1-1	1.0	269	2	0	2	0
MDSM	DLG1-2	0.875	269	3	0	3	0
MDSM	MPDZ-10	1.0	2534	1	0	4	0
MDSM	ERBB2IP-1	1.0	825	0	0	2	0
MDSM	DLG3-2	0.875	269	1	0	2	0
MDSM	LIN7B-1	1.0	165	2	0	2	0
MDSM	DLG4-1	0.9375	269	2	0	2	0
MDSM	DLG4-2	0.8125	269	2	0	2	0
MDSM	PDZK1-1	0.9375	11	0	0	1	0

MDSM	MLLT4-1	0.6875	285	1	0	6	0
MDSM	MAGI3-1	0.6875	1070	0	0	1	0
MDSM	MAGI2-2	0.75	1070	0	0	1	0
MDSM	SNTG1-1	0.875	613	1	0	1	0
Method	Domain	NN Sim	Num Predicted	#TP	#FP	#Valid Positives	#Valid Negatives
Additive	DLG1-1	1.0	2094	2	0	2	0
Additive	DLG1-2	1.0	2241	3	0	3	0
Additive	MPDZ-10	1.0	52	0	0	4	0
Additive	ERBB2IP-1	1.0	395	0	0	2	0
Additive	DLG3-2	1.0	2241	1	0	2	0
Additive	LIN7B-1	1.0	2734	1	0	2	0
Additive	DLG4-1	0.9375	1960	2	0	2	0
Additive	DLG4-2	0.9375	2041	2	0	2	0
Additive	PDZK1-1	0.8125	0	0	0	1	0
Additive	MLLT4-1	0.6875	93	1	0	6	0
Additive	MAGI3-1	1.0	1846	0	0	1	0
Additive	MAGI2-2	1.0	2406	1	0	1	0
Additive	SNTG1-1	1.0	1723	1	0	1	0
Method	Domain	NN Sim	Num Predicted	#TP	#FP	#Valid Positives	#Valid Negatives
PWM	DLG1-1	1.0	412	1	0	2	0
PWM	DLG1-2	1.0	412	3	0	3	0
PWM	MPDZ-10	1.0	412	4	0	4	0
PWM	ERBB2IP-1	1.0	412	2	0	2	0
PWM	DLG3-2	1.0	412	1	0	2	0
PWM	LIN7B-1	1.0	412	2	0	2	0
PWM	DLG4-1	0.9375	412	1	0	2	0
PWM	DLG4-2	0.9375	412	2	0	2	0
PWM	PDZK1-1	0.8125	412	1	0	1	0
PWM	MLLT4-1	0.6875	412	2	0	6	0
PWM	MAGI3-1	1.0	412	0	0	1	0
PWM	MAGI2-2	1.0	412	0	0	1	0
PWM	SNTG1-1	1.0	412	1	0	1	0

Worm

The worm proteome was scanned to predict interactions for 6 worm PDZ domains with positive and negative interactions from protein microarray experiments [3]. In total, 19,864 unique transcript tails of length five out of 27,533 transcripts corresponding to

20,158 genes in the worm proteome were scanned (defined by genome assembly Ensembl:WS200.56) [7].

Table S6. The following table summarizes the worm proteome scanning results for the SVM, MDSM, Additive and PWM predictors

Method	Domain	NN Sim	Num Predicted	#TP	#FP	#Valid Positives	#Valid Negatives
SVM	DLG1-1	0.8125	44	1	1	4	18
SVM	DLG1-3	0.9375	87	4	1	7	15
SVM	DSH-1	0.8125	14	0	0	11	4
SVM	LIN7-1	1.0	159	3	1	11	11
SVM	MPZ1-6	0.6875	144	4	0	18	4
SVM	STN2-1	0.8125	256	3	0	8	14
Method	Domain	NN Sim	Num Predicted	#TP	#FP	#Valid Positives	#Valid Negatives
MDSM	DLG1-1	0.75	110	1	1	4	18
MDSM	DLG1-3	0.9375	168	4	1	7	15
MDSM	DSH-1	0.8125	2598	3	0	11	4
MDSM	LIN7-1	1.0	61	1	0	11	11
MDSM	MPZ1-6	0.6875	85	0	0	18	4
MDSM	STN2-1	0.8125	200	3	1	8	14
Method	Domain	NN Sim	Num Predicted	#TP	#FP	#Valid Positives	#Valid Negatives
Additive	DLG1-1	0.8125	730	2	4	4	18
Additive	DLG1-3	0.9375	864	4	3	7	15
Additive	DSH-1	0.8125	79	0	0	11	4
Additive	LIN7-1	1.0	1177	7	2	11	11
Additive	MPZ1-6	0.6875	713	3	0	18	4
Additive	STN2-1	0.8125	1086	4	2	8	14
Method	Domain	NN Sim	Num Predicted	#TP	#FP	#Valid Positives	#Valid Negatives
PWM	DLG1-1	0.8125	199	2	4	4	18
PWM	DLG1-3	0.9375	199	1	2	7	15
PWM	DSH-1	0.8125	199	1	0	11	4
PWM	LIN7-1	1.0	199	3	2	11	11
PWM	MPZ1-6	0.6875	199	3	1	18	4
PWM	STN2-1	0.8125	199	4	2	8	14

Fly

The fly proteome was scanned to predict interactions for 7 fly PDZ domains with positive and negative interactions from protein microarray experiments [3]. In total, 14,691 unique transcript tails of length five out of 21,309 transcripts corresponding to 20,158 genes were scanned (defined by genome assembly Ensembl:BDGP5.13.56) [7].

Table S7. The following table summarizes the fly proteome scanning results for the SVM, MDSM, Additive and PWM predictors

Method	Domain	NN Sim	Num Predicted	#TP	#FP	#Valid Positives	#Valid Negatives
SVM	MAGI-4	0.8125	92	2	3	2	17
SVM	DLG1-1	0.9375	112	4	0	4	15
SVM	DSH-1	0.9375	49	0	0	3	16
SVM	LAP4-2	0.875	30	3	1	5	14
SVM	LAP4-3	0.75	8	2	0	8	11
SVM	PAR6-1	1.0	0	0	0	1	18
SVM	PATJ-2	0.8125	184	0	0	7	12
Method	Domain	NN Sim	Num Predicted	#TP	#FP	#Valid Positives	#Valid Negatives
MDSM	MAGI-4	0.8125	192	0	0	2	17
MDSM	DLG1-1	0.9375	76	2	2	4	15
MDSM	DSH-1	0.9375	1641	2	3	3	16
MDSM	LAP4-2	0.875	8	0	0	5	14
MDSM	LAP4-3	0.75	95	4	1	8	11
MDSM	PAR6-1	1.0	3	0	0	1	18
MDSM	PATJ-2	0.625	5	1	0	7	12
Method	Domain	NN Sim	Num Predicted	#TP	#FP	#Valid Positives	#Valid Negatives
Additive	MAGI-4	0.8125	843	2	6	2	17
Additive	DLG1-1	0.9375	849	4	3	4	15
Additive	DSH-1	0.9375	98	0	0	3	16
Additive	LAP4-2	0.875	307	4	1	5	14
Additive	LAP4-3	0.75	300	3	0	8	11
Additive	PAR6-1	1.0	18	0	0	1	18
Additive	PATJ-2	0.625	30	0	0	7	12
Method	Domain	NN Sim	Num Predicted	#TP	#FP	#Valid Positives	#Valid Negatives
PWM	MAGI-4	0.8125	147	0	3	2	17

PWM	DLG1-1	0.9375	147	4	2	4	15
PWM	DSH-1	0.9375	147	1	3	3	16
PWM	LAP4-2	0.875	147	5	3	5	14
PWM	LAP4-3	0.75	147	4	2	8	11
PWM	PAR6-1	1.0	147	0	0	1	18
PWM	PATJ-2	0.8125	147	0	1	7	12

G. Binding sequence similarity calculation

The distance between two domain binding site sequences a and b of the same length n is calculated as the Hamming distance between the two sequences (Equation 1). The sequence similarity between the two sequences is therefore 1.0 minus the Hamming distance (Equation 2):

$$\text{Distance}_{seq}(a,b) = \frac{\sum_{i=n}^{n} \text{match}(a_i,b_i)}{n - \sum_{i=1}^{n} \text{gap}(a_i,b_i)} \qquad (1)$$

$$\text{Similarity}_{seq}(a,b) = 1.0 - \text{Distance}_{seq}(a,b) \qquad (2)$$

where match is 1 if a_i=b_i , otherwise 0, gap equals 1 if a_i or b_i is a gap, otherwise 0.

H. Binding specificity similarity calculation

The distance between two PWMs a and b is the normalized Euclidean distance (Equation 3). The similarity between two profiles is therefore 1 minus the distance:

$$\text{Distance}_{PWM}(a,b) = \frac{1}{\sqrt{2}} \sum_{i=1}^{w} \sqrt{\sum_{L \in \{20aa's\}} (a_{i,L} - b_{i,L})^2} \qquad (3)$$

$$\text{Similarity}_{PWM}(a,b) = 1.0 - \text{Distance}_{PWM}(a,b) \qquad (4)$$

where w is the number of columns in the PWM. This metric is normalized such that 0 represents perfectly similar PWMs and 1 represents perfectly dissimilar PWMs.

I. Comparison of genomic phage display and predicted sequence logos

Genomic phage display sequence logos were created by scanning the human proteome for the top 1% of binders using the PWMs created with optimal phage display binders. The optimal and genomic phage display sequence logos were then compared to the corresponding SVM predicted sequence logos.

Figure S2. The following is a comparison of the optimal phage display and genomic phage display sequence logos compared to the corresponding predicted SVM sequence logos for the last five terminal binding positions. Only the four human PDZ domains from Figure 4 of the paper were compared.

Domain Name	NN Sim	Optimal	Genomic	SVM Predicted	Optimal Profile Sim	Genomic Profile Sim
DLG1-2 Human	1	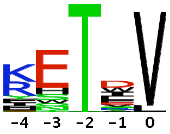	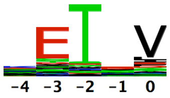	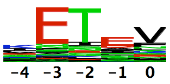	0.751	0.886
DLG3-2 Human	1	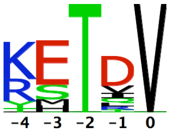	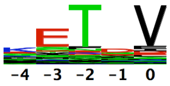	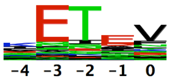	0.682	0.86
MLLT4-1 Human	0.69	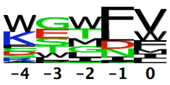	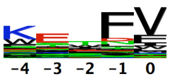	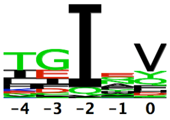	0.62	0.624
PDZK1-1 Human	0.81	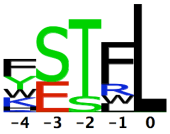	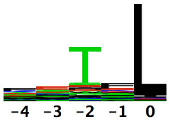	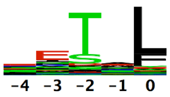	0.691	0.851

J. Comparison of the performance of MDSM and SVM trained using only microarray data

To more directly compare the MDSM and SVM, we trained an SVM with only mouse microarray data and compared the performance of the predictors.

Figure S3. The following is a comparison of MDSM and SVM performance evaluated using F1 measures and FPRs for 13 human (blue), 6 worm (green) and 7 fly (black) PDZ domains. The median is denoted by the red circle. No FPRs were calculated for human predictions since there are no negative human validation interaction data. Both predictors were trained using microarray training data only.

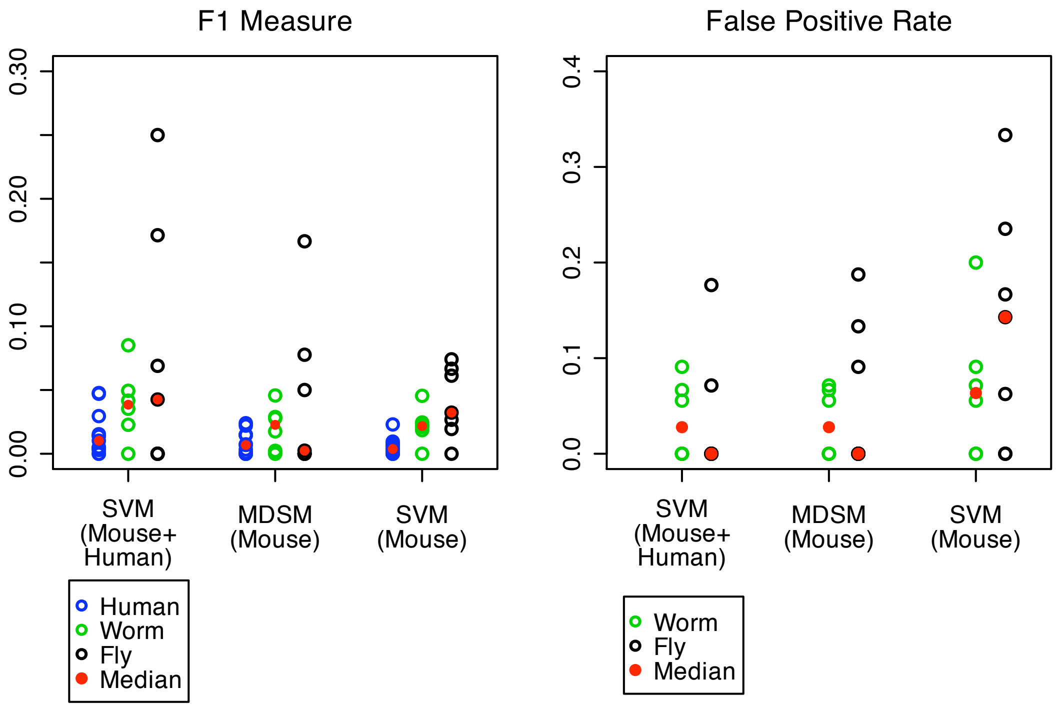

K. Protein protein interaction evidence to support PDZ domain peptide predictions

Physical human protein protein interactions (PPIs) were collected from eight interaction databases (BIND, BioGRID, CORUM, DIP, HPRD, IntAct, MINT and MPPI) through

the iRefIndex database [8]. Only interactions annotated with UniProt ids from UniProtKB/Swiss-Prot were used (since the corresponding sequences were manually annotated and reviewed). A PPI was counted as corresponding to a domain peptide interaction prediction if the protein containing the domain was found in iRefIndex to interact with the protein containing the peptide. To test the significance of the number of predictions found to be in iRefIndex for a given domain, a Fisher's exact test was performed and asked whether the observed number predictions could be achieved at random. In total, 213 human PDZ domains with PPIs in iRefIndex were analyzed. The SVM predicted interactions for 192 domains with 75 domains having predictions corresponding to at least one iRefIndex interaction. The SVM did not make predictions for the remaining 21 domains.

Table S8. The following table lists the identities of the 75 human PDZ domains whose proteome predicts corresponded to at least one protein-protein interacion from iRefIndex. UniProt Domain Name is the name of the domain using the UniProt protein name. UniProt Domain Sequence Positions are the start and end positions of the domain sequence along the UniProt protein sequence. UniProt ID is the identifier of the UniProt protein. Tonikian Domain Name is the name of the domain used in Tonikian et al.

UniProt Domain Name	UniProt Domain Sequence Positions	UniProt ID	Tonikian Domain Name
ARHGC-1	72-151	Q9NZN5	
GIPC1-1	133-213	O14908	
LIN7B-1	93-175	Q9HAP6	
MAGI2-1	17-101	Q86UL8	
MAGI2-2	426-510	Q86UL8	
MAGI2-4	778-860	Q86UL8	
MAGI2-5	920-1010	Q86UL8	

MAGI2-3	605-683	Q86UL8	
MAGI2-6	1147-1229	Q86UL8	
MAST2-1	967-1055	Q9Y2H9	
MPP3-1	137-212	Q13368	
NHRF1-1	14-94	O14745	
NHRF1-2	154-234	O14745	
NHRF3-2	134-215	Q5T2W1	
NHRF3-4	378-458	Q5T2W1	
NHRF3-3	243-323	Q5T2W1	
NHRF4-1	115-196	Q86UT5	
NHRF4-3	329-412	Q86UT5	
PDLI1-1	3-85	O00151	
PDZ11-1	47-129	Q5EBL8	
PDZD2-2	334-419	O15018	
PTN3-1	510-582	P26045	
RGS12-1	22-98	O14924	
RGS3-1	299-376	P49796	
SHAN1-1	663-757	Q9Y566	
SHAN2-1	247-341	Q9UPX8	
SNTB1-1	112-195	Q13884	
SNTB2-1	115-198	Q13425	
SNTG1-1	57-140	Q9NSN8	
SNTG2-1	73-156	Q9NY99	
SYJ2B-1	13-100	P57105	
APBA3-2	485-560	O96018	
DLG3-1	130-217	Q92796	
DLG3-3	379-465	Q92796	
DLG4-2	160-246	P78352	
DLG4-1	65-151	P78352	
INADL-8	1437-1520	Q8NI35	
MPDZ-8	1350-1433	O75970	
NHRF2-1	11-90	Q15599	
PARD3-3	590-680	Q8TEW0	PARD3-3
MPDZ-4	565-630	O75970	MPDZ-4
MPDZ-7	1151-1239	O75970	MPDZ-7
MPDZ-10	1629-1708	O75970	MPDZ-10
MPDZ-13	1959-2038	O75970	MPDZ-13
NHRF2-2	151-227	Q15599	SLC9A3R2-2
DLG4-3	313-390	P78352	DLG4-3
MPDZ-2	257-333	O75970	MPDZ-2
SCRIB-4	1110-1194	Q14160	
ZO2-1	33-120	Q9UDY2	
DLG1-1	224-307	Q12959	DLG1-1
DLG1-2	319-402	Q12959	DLG1-2
DLG1-3	466-543	Q12959	DLG1-3

DLG3-2	226-309	Q92796	DLG3-2
MAGI1-2	472-554	Q96QZ7	
MAGI1-3	634-719	Q96QZ7	MAGI1-2
MAGI1-4	813-895	Q96QZ7	
MAGI1-6	1124-1206	Q96QZ7	
MAGI3-4	751-831	Q5TCQ9	MAGI3-3
MAGI3-5	876-963	Q5TCQ9	
MAGI3-5	1046-1128	Q5TCQ9	
PTN13-2	368-1449	Q12923	PTPN13-2
SCRIB-1	728-811	Q14160	SCRIB-1
SCRIB-2	862-947	Q14160	SCRIB-2
SCRIB-3	1004-1093	Q14160	
DLG2-2	193-279	Q15700	
DLG2-1	98-184	Q15700	
DLG2-3	421-501	Q15700	
LAP2-1	1323-1406	Q96RT1	ERBB2IP-1
LRRC7-1	1448-1531	Q96NW7	LRRC7-1
CSKP-1	490-566	O14936	CASK-1
AFAD-1	1009-1087	P55196	MLLT4-1
SNTA1-1	87-166	Q13424	SNTA1-1
MAGI3-2	435-517	Q5TCQ9	
MAGI3-3	603-679	Q5TCQ9	
NHRF3-1	9-86	Q5T2W1	PDZK1-1

Table S9. The following table lists the number of predicted interactions that correspond to protein-protein interactions in iRefIndex for 75 human PDZ domains. UniProt Domain Name is the name of the domain using the UniProt protein name.

UniProt Domain Name	# iRefIndex PPIs predicted	# iRefIndex PPIs	*p*-value
ARHGC-1	1	14	0.566
GIPC1-1	4	42	7.76e-06
LIN7B-1	1	11	0.107
MAGI2-1	1	10	0.124
MAGI2-2	2	10	0.0117
MAGI2-4	1	10	0.0325
MAGI2-5	1	10	0.0344
MAGI2-3	1	10	0.122
MAGI2-6	1	10	0.0952
MAST2-1	2	6	0.0017
MPP3-1	1	1	0.000631

NHRF1-1	15	57	7.45e-15
NHRF1-2	24	57	1.74e-14
NHRF3-2	1	24	0.0763
NHRF3-4	3	24	0.00141
NHRF3-3	8	24	3.08e-06
NHRF4-1	1	5	0.0408
NHRF4-3	3	5	0.000206
PDLI1-1	1	14	0.0748
PDZ11-1	1	4	0.0307
PDZD2-2	1	5	0.123
PTN3-1	1	5	0.0861
RGS12-1	4	19	0.000715
RGS3-1	3	11	0.026
SHAN1-1	2	21	0.0913
SHAN2-1	1	13	0.364
SNTB1-1	4	14	9.74e-06
SNTB2-1	3	20	0.00105
SNTG1-1	1	12	0.181
SNTG2-1	1	1	0.0114
SYJ2B-1	3	5	5.71e-05
APBA3-2	1	7	0.00289
DLG3-1	9	48	3.98e-11
DLG3-3	7	48	1.95e-07
DLG4-2	14	130	2.88e-11
DLG4-1	13	130	7.37e-12
INADL-8	1	15	0.0653
MPDZ-8	1	9	0.0141
NHRF2-1	12	44	2.48e-12
PARD3-3	1	26	0.0311
MPDZ-4	2	9	0.0081
MPDZ-7	1	9	0.027
MPDZ-10	4	9	6.53e-08
MPDZ-13	1	9	0.0137
NHRF2-2	15	44	2.33e-11
DLG4-3	13	130	1.41e-10
MPDZ-2	1	9	0.0591
SCRIB-4	1	11	0.0534
ZO2-1	1	11	0.0844
DLG1-1	13	83	1.98e-14
DLG1-2	14	83	5.21e-14
DLG1-3	10	83	3.09e-09
DLG3-2	9	48	6.6e-10
MAGI1-2	4	24	0.00014
MAGI1-3	3	24	0.000176
MAGI1-4	2	24	0.000724

MAGI1-6	6	24	4.54e-05
MAGI3-4	1	12	0.0426
MAGI3-5	1	12	0.0256
MAGI3-6	1	12	0.31
PTN13-2	1	23	0.111
SCRIB-1	1	11	0.0357
SCRIB-2	1	11	0.0292
SCRIB-3	1	11	0.161
DLG2-2	8	41	4.28e-09
DLG2-1	8	41	3.53e-10
DLG2-3	6	41	1.46e-06
LAP2-1	2	33	0.00203
LRRC7-1	2	13	0.000731
CSKP-1	3	53	0.0396
AFAD-1	1	58	0.0495
SNTA1-1	4	28	9.53e-05
MAGI3-2	5	12	1.31e-05
MAGI3-3	1	12	0.0199
NHRF3-1	4	24	0.000272

L. GO biological process term enrichment

GO biological process term enrichment analysis was performed to determine statistically overrepresented annotations in the genes of predicted binders for the PDZ domains used in proteome scanning tests. The hypergeometric test was used to compute a *p*-value to assess GO term enrichment for a set of predicted genes. Since this results in testing the significance of all GO terms in the given set of genes in a single analysis, multiple testing correction was performed using the Benjamini and Hochberg False Discovery Rate (FDR) correction with a significance level of 0.05. The BiNGO (Biological Network Gene Ontology tool) [9] software library was used. Only manually annotated GO terms were used.

Table S10. The following table lists the enriched GO biological process terms in genes of predicted binders for 13 human PDZ domains used for proteome scanning. GO ID is the GO process term identifier, *p*-value is the hypergeometric test statistic corrected for multiple testing, Description is the GO term description. GO terms are ordered by increasing *p*-value. Only GO terms with $p < 0.05$ are displayed. Domains with no terms satisfying this cutoff are indicated by an asterisk and only the top 10 GO terms are displayed.

DLG1-1-Human		
GO ID	*p*-value	Description
6813	2.658E-3	potassium ion transport
30001	2.658E-3	metal ion transport
6811	3.062E-3	ion transport
6812	3.481E-3	cation transport
15672	8.531E-3	monovalent inorganic cation transport
DLG1-2-Human		
GO ID	*p*-value	Description
6811	2.774E-4	ion transport
6813	2.167E-3	potassium ion transport
6812	5.264E-3	cation transport
30001	5.264E-3	metal ion transport
6810	1.151E-2	transport
15672	1.685E-2	monovalent inorganic cation transport
51234	2.034E-2	establishment of localization
DLG3-2-Human		
GO ID	*p*-value	Description
6811	2.774E-4	ion transport
6813	2.167E-3	potassium ion transport
6812	5.264E-3	cation transport
30001	5.264E-3	metal ion transport
6810	1.151E-2	transport
15672	1.685E-2	monovalent inorganic cation transport
51234	2.034E-2	establishment of localization
DLG4-1-Human		
GO ID	*p*-value	Description
6813	2.658E-3	potassium ion transport
30001	2.658E-3	metal ion transport
6811	3.062E-3	ion transport
6812	3.481E-3	cation transport

15672	8.531E-3	monovalent inorganic cation transport
DLG4-2-Human		
GO ID	*p*-value	Description
6811	2.774E-4	ion transport
6813	2.167E-3	potassium ion transport
6812	5.264E-3	cation transport
30001	5.264E-3	metal ion transport
6810	1.151E-2	transport
15672	1.685E-2	monovalent inorganic cation transport
51234	2.034E-2	establishment of localization
ERBB2IP-1-Human *		
GO ID	*p*-value	Description
32581	2.557E-1	ER-dependent peroxisome biogenesis
16557	2.557E-1	peroxisome membrane biogenesis
45046	2.557E-1	protein import into peroxisome membrane
55114	2.557E-1	oxidation reduction
6338	2.557E-1	chromatin remodeling
7155	2.557E-1	cell adhesion
22610	2.557E-1	biological adhesion
51016	2.557E-1	barbed-end actin filament capping
51693	2.557E-1	actin filament capping
15917	2.557E-1	aminophospholipid transport
LIN7B-1-Human *		
GO ID	*p*-value	Description
6811	1.414E-1	ion transport
35176	1.414E-1	social behavior
6813	1.414E-1	potassium ion transport
6812	1.414E-1	cation transport
30001	1.414E-1	metal ion transport
30516	1.414E-1	regulation of axon extension
32927	1.414E-1	positive regulation of activin receptor signaling pathway
51705	1.414E-1	behavioral interaction between organisms
1935	1.414E-1	endothelial cell proliferation
50808	1.414E-1	synapse organization and biogenesis
MAGI2-2-Human *		
GO ID	*p*-value	Description
7389	3.909E-1	pattern specification process
35176	3.909E-1	social behavior
6812	3.909E-1	cation transport
6810	3.909E-1	transport
7264	3.909E-1	small GTPase mediated signal transduction
6813	3.909E-1	potassium ion transport
51234	3.909E-1	establishment of localization
51179	3.909E-1	localization

32927	3.909E-1	positive regulation of activin receptor signaling pathway
51705	3.909E-1	behavioral interaction between organisms
MAGI3-1-Human		
GO ID	*p*-value	Description
6813	1.458E-2	potassium ion transport
51234	1.768E-2	establishment of localization
6810	1.768E-2	transport
6811	1.768E-2	ion transport
51179	1.768E-2	localization
MLLT4-1-Human *		
GO ID	*p*-value	Description
33081	5.388E-2	regulation of T cell differentiation in the thymus
46620	5.388E-2	regulation of organ growth
303	5.388E-2	response to superoxide
45541	5.388E-2	negative regulation of cholesterol biosynthetic process
48538	5.388E-2	thymus development
45939	5.388E-2	negative regulation of steroid metabolic process
45540	5.388E-2	regulation of cholesterol biosynthetic process
1890	5.388E-2	placenta development
305	5.388E-2	response to oxygen radical
50810	7.339E-2	regulation of steroid biosynthetic process
MPDZ-10-Human *		
GO ID	*p*-value	Description
6813	7.25E-2	potassium ion transport
1508	1.822E-1	regulation of action potential
30001	1.822E-1	metal ion transport
15672	1.822E-1	monovalent inorganic cation transport
6342	1.822E-1	chromatin silencing
31507	1.822E-1	heterochromatin formation
42391	1.822E-1	regulation of membrane potential
45814	1.822E-1	negative regulation of gene expression, epigenetic
6812	1.822E-1	cation transport
19226	1.822E-1	transmission of nerve impulse
PDZK1-1-Human		
GO ID	*p*-value	Description
6811	2.389E-4	ion transport
45494	5.702E-3	photoreceptor cell maintenance
SNTG1-1-Human		
GO ID	*p*-value	Description
6810	2.251E-2	transport
51234	2.251E-2	establishment of localization
46942	3.625E-2	carboxylic acid transport
6813	3.625E-2	potassium ion transport

15849	3.625E-2	organic acid transport

Table S11. The following table lists the enriched GO biological process terms in genes of predicted binders for 6 worm PDZ domains used for proteome scanning. GO ID is the GO process term identifier, *p*-value is the hypergeometric test statistic corrected for multiple testing, Description is the GO term description. GO terms are ordered by increasing *p*-value. Only GO terms with $p < 0.05$ are displayed. Domains with no terms satisfying this cutoff are indicated by an asterisk and only the top 10 GO terms are displayed.

DLG1-1-Worm		
GO ID	*p*-value	Description
35046	5.259E-3	pronuclear migration
7097	5.259E-3	nuclear migration
7338	5.259E-3	single fertilization
51647	5.259E-3	nucleus localization
40023	5.259E-3	establishment of nucleus localization
9566	5.259E-3	fertilization
51656	1.647E-2	establishment of organelle localization
51640	1.647E-2	organelle localization
51649	2.844E-2	establishment of localization in cell
51641	2.844E-2	cellular localization
48755	2.844E-2	branching morphogenesis of a nerve
1763	2.844E-2	morphogenesis of a branching structure
51179	3.099E-2	localization
7166	3.099E-2	cell surface receptor linked signal transduction
8039	3.099E-2	synaptic target recognition
33673	3.099E-2	negative regulation of kinase activity
7219	3.099E-2	Notch signaling pathway
43407	3.099E-2	negative regulation of MAP kinase activity
6469	3.099E-2	negative regulation of protein kinase activity
43086	3.099E-2	negative regulation of catalytic activity
51348	3.099E-2	negative regulation of transferase activity
8543	3.099E-2	fibroblast growth factor receptor signaling pathway
7154	3.31E-2	cell communication
19953	3.31E-2	sexual reproduction
51234	4.794E-2	establishment of localization

7052	4.927E-2	mitotic spindle organization and biogenesis
43405	4.992E-2	regulation of MAP kinase activity
8151	4.992E-2	cellular process
7051	4.992E-2	spindle organization and biogenesis
50808	4.992E-2	synapse organization and biogenesis
51338	4.992E-2	regulation of transferase activity
31344	4.992E-2	regulation of cell projection organization and biogenesis
43549	4.992E-2	regulation of kinase activity
45859	4.992E-2	regulation of protein kinase activity
DLG1-3-Worm		
GO ID	*p*-value	Description
35046	2.409E-2	pronuclear migration
7097	2.409E-2	nuclear migration
7338	2.409E-2	single fertilization
51647	2.409E-2	nucleus localization
40023	2.409E-2	establishment of nucleus localization
9566	2.409E-2	fertilization
DSH-1-Worm *		
GO ID	*p*-value	Description
40017	2.168E-1	positive regulation of locomotion
40012	2.168E-1	regulation of locomotion
40015	6.368E-1	negative regulation of multicellular organism growth
51241	6.368E-1	negative regulation of multicellular organismal process
45926	6.368E-1	negative regulation of growth
40035	6.413E-1	hermaphrodite genitalia development
48806	6.413E-1	genitalia development
7548	6.413E-1	sex differentiation
3006	6.413E-1	reproductive developmental process
48513	6.413E-1	organ development
LIN7-1-Worm *		
GO ID	*p*-value	Description
50793	1.159E-1	regulation of developmental process
51656	1.159E-1	establishment of organelle localization
51640	1.159E-1	organelle localization
40028	1.159E-1	regulation of vulval development
35046	1.159E-1	pronuclear migration
226	1.159E-1	microtubule cytoskeleton organization and biogenesis
7097	1.159E-1	nuclear migration
7338	1.159E-1	single fertilization
51647	1.159E-1	nucleus localization
40023	1.159E-1	establishment of nucleus localization

MPZ1-6-Worm *		
GO ID	*p*-value	Description
6937	1.694E-1	regulation of muscle contraction
22604	1.694E-1	regulation of cell morphogenesis
7154	1.694E-1	cell communication
50793	1.694E-1	regulation of developmental process
8151	1.694E-1	cellular process
10248	1.694E-1	establishment and/or maintenance of transmembrane electrochemical gradient
45750	1.694E-1	positive regulation of S phase of mitotic cell cycle
48755	1.694E-1	branching morphogenesis of a nerve
1763	1.694E-1	morphogenesis of a branching structure
51179	1.694E-1	localization
STN2-1-Worm *		
GO ID	*p*-value	Description
50793	2.807E-1	regulation of developmental process
22604	2.807E-1	regulation of cell morphogenesis
10248	2.807E-1	establishment and/or maintenance of transmembrane electrochemical gradient
45750	2.807E-1	positive regulation of S phase of mitotic cell cycle
7166	2.807E-1	cell surface receptor linked signal transduction
45167	2.807E-1	asymmetric protein localization during cell fate commitment
51656	2.807E-1	establishment of organelle localization
51640	2.807E-1	organelle localization
35046	2.807E-1	pronuclear migration
51179	2.807E-1	localization

Table S12. The following table lists the enriched GO biological process terms in genes of predicted binders for 6 fly PDZ domains used for proteome scanning (with SVM predictions). GO ID is the GO process term identifier, *p*-value is the hypergeometric test statistic corrected for multiple testing, Description is the GO term description. GO terms are ordered by increasing *p*-value. Only GO terms with $p < 0.05$ are displayed. Domains with no terms satisfying this cutoff are indicated by an asterisk and only the top 10 GO terms are displayed.

DLG1-1-Fly		
GO ID	*p*-value	Description

16337	6.252E-4	cell-cell adhesion
7156	2.479E-2	homophilic cell adhesion
7155	2.479E-2	cell adhesion
22610	3.779E-2	biological adhesion
DSH-1-Fly *		
GO ID	*p*-value	Description
7476	6.294E-2	imaginal disc-derived wing morphogenesis
7472	6.294E-2	wing disc morphogenesis
48082	6.294E-2	regulation of adult chitin-containing cuticle pigmentation
48079	6.294E-2	regulation of cuticle pigmentation
7480	6.294E-2	imaginal disc-derived leg morphogenesis
35114	6.294E-2	imaginal disc-derived appendage morphogenesis
48737	6.294E-2	imaginal disc-derived appendage development
35107	6.294E-2	appendage morphogenesis
35220	6.294E-2	wing disc development
48736	6.294E-2	appendage development
LAP4-2-Fly *		
GO ID	*p*-value	Description
30038	1.47E-1	contractile actin filament bundle formation
16337	1.47E-1	cell-cell adhesion
48150	1.47E-1	behavioral response to ether
6207	1.47E-1	'de novo' pyrimidine base biosynthetic process
45472	1.47E-1	response to ether
34404	1.47E-1	nucleobase, nucleoside and nucleotide biosynthetic process
46112	1.47E-1	nucleobase biosynthetic process
19856	1.47E-1	pyrimidine base biosynthetic process
48644	1.47E-1	muscle morphogenesis
6206	1.602E-1	pyrimidine base metabolic process
LAP4-3-Fly *		
GO ID	*p*-value	Description
14902	6.15E-2	myotube differentiation
768	6.15E-2	syncytium formation by plasma membrane fusion
6949	6.15E-2	syncytium formation
6947	6.15E-2	plasma membrane fusion
7520	6.15E-2	myoblast fusion
48627	6.15E-2	myoblast development
48628	6.15E-2	myoblast maturation
45445	6.15E-2	myoblast differentiation
6944	6.15E-2	membrane fusion
48469	6.15E-2	cell maturation
MAGI-Fly *		
GO ID	*p*-value	Description
16339	2.161E-1	calcium-dependent cell-cell adhesion

7413	2.161E-1	axonal fasciculation
7155	2.161E-1	cell adhesion
7409	2.161E-1	axonogenesis
44265	2.161E-1	cellular macromolecule catabolic process
48675	2.161E-1	axon extension
22610	2.161E-1	biological adhesion
6393	2.161E-1	termination of mitochondrial transcription
6390	2.161E-1	transcription from mitochondrial promoter
8040	2.161E-1	axon guidance
PATJ-2-Fly *		
GO ID	*p*-value	Description
48133	1.978E-1	male germ-line stem cell division
42078	3.139E-1	germ-line stem cell division
17145	3.139E-1	stem cell division
45786	3.139E-1	negative regulation of cell cycle
16199	3.139E-1	axon midline choice point recognition
7346	3.139E-1	regulation of mitotic cell cycle
46864	3.139E-1	isoprenoid transport
45910	3.139E-1	negative regulation of DNA recombination
46866	3.139E-1	tetraterpenoid transport
46865	3.139E-1	terpenoid transport

References

1. **LIBSVM: a library for support vector machines** [http://www.csie.ntu.edu.tw/~cjlin/libsvm]

2. Tonikian R, Zhang Y, Sazinsky SL, Currell B, Yeh JH, Reva B, Held HA, Appleton BA, Evangelista M, Wu Y, et al: **A specificity map for the PDZ domain family.** *PLoS Biol* 2008, **6:**e239.

3. Chen JR, Chang BH, Allen JE, Stiffler MA, MacBeath G: **Predicting PDZ domain-peptide interactions from primary sequences.** *Nat Biotechnol* 2008, **26:**1041-1045.

4. Skelton NJ, Koehler MF, Zobel K, Wong WL, Yeh S, Pisabarro MT, Yin JP, Lasky LA, Sidhu SS: **Origins of PDZ domain ligand specificity. Structure determination and mutagenesis of the Erbin PDZ domain.** *J Biol Chem* 2003, **278:**7645-7654.

5. Stiffler MA, Chen JR, Grantcharova VP, Lei Y, Fuchs D, Allen JE, Zaslavskaia LA, MacBeath G: **PDZ domain binding selectivity is optimized across the mouse proteome.** *Science* 2007, **317:**364-369.

6. Beuming T, Skrabanek L, Niv MY, Mukherjee P, Weinstein H: **PDZBase: a protein-protein interaction database for PDZ-domains.** *Bioinformatics* 2005, **21:**827-828.

7. Hubbard TJP, Aken BL, Ayling S, Ballester B, Beal K, Bragin E, Brent S, Chen Y, Clapham P, Clarke L, et al: **Ensembl 2009.** *Nucleic Acids Res* 2009, **37:**D690-D697.

8. Razick S, Magklaras G, Donaldson IM: **iRefIndex: A consolidated protein interaction database with provenance.** *BMC Bioinformatics* 2008, **9:**405.

9. Maere S, Heymans K, Kuiper M: **BiNGO: a Cytoscape plugin to assess overrepresentation of Gene Ontology categories in Biological Networks.** *Bioinformatics* 2005, **21:**3448-3449.
